# Youth development and spatial configurations: socio-spatial inequalities in Palestinian refugee camps in Lebanon

**DOI:** 10.3389/fsoc.2023.1192511

**Published:** 2023-11-15

**Authors:** Yara El-Zakka, Jasmin Lilian Diab

**Affiliations:** ^1^Institute for Migration Studies, School of Arts and Sciences, Lebanese American University, Beirut, Lebanon; ^2^Department of Social and Education Sciences, School of Arts and Sciences, Lebanese American University, Beirut, Lebanon

**Keywords:** Palestinian, inequality, development, camps, youth, socio-spatial

## Abstract

This article explores the intersectional fields of displacement and architecture by examining the interrelationship between youth development and spatial configurations in Palestinian refugee camps in Lebanon, more specifically in Ein El Hilweh Camp, South of the country. It aims at understanding how spatial configurations can alter human behavior, and the ways in which societal reform can take place in an urban context. Through taking Ein El Hilweh Camp as a case study and field interviews with experts working and residing in the Ein El-Hilweh Camp, this article asserts that youth development in Palestinian Camps in Lebanon is hindered by the dire conditions of spatial configurations in the camps and their geopolitics. It also asserts that in the presence of fostered youth protection and capacity and skills building, youth engagement and participation in the modification of their spaces act as essential drivers of change which contribute to the reduction of urban poverty and to the development of urban strategies that can sustain their development and provide an incubating environment for them to grow during the stages of their youth and beyond. Moreover, this article suggests that the primary factor contributing to the hindered situation of Palestinian refugees in Lebanon is the condition of permanent temporariness imposed by the state. The reduction of socio-spatial inequalities is temporary unless the integration of Palestinian refugees is fostered and their accessibility to social, economic, and civil rights, as well as the right to the city is granted.

## 1. Introduction

The intersectionality between social differences – ultimately developing into inequalities with modern urbanization – and the spaces they occupy is capable of generating urban exclusions which hinder the equal and sustainable development of societies. The apex of these exclusions is present in the settlements of the displaced populations that are mostly engendered by conflict, specifically, as the notion of displacement is quite often portrayed as a transitory state of being. More specifically, these settlements are kept with transitory infrastructures and a spatial configuration with defined boundaries which reinforces its inhabitants’ segregation from the local community, prohibiting them from engaging in the local economy, and consequently rendering them dependent on aid agencies for their basic means of survival. Youth, who are a vital asset for reaching safe, resilient, and sustainable cities are among the most affected by these socio-spatial inequalities and demonstrate a reciprocal relationship with the urban landscape.

This study explores the intersectional fields of displacement and architecture by examining the interrelationship between youth development and spatial configurations in Palestinian refugee camps^1^ in Lebanon, taking Ein El Hilweh Camp in the South of Lebanon as a case study. This article aims at understanding how spatial configurations can alter human behavior, and the ways in which societal reform can take place in an urban context. While space modifies and is modified by anyone who occupies it, this research will be narrowed down to Youth, who are presupposed to be the primary force of change and refinement in society.

This study involves a profound and contextual understanding of Palestinian refugee camps in Lebanon by situating the spatial configurations within the temporality imposed by the state. It will explicate on the different variables producing socio-spatial inequalities, explicitly the geographic politics of the camps, their spatial configurations, and the underdevelopment of infrastructure, services, and facilities in these camps. The implications of the socio-spatial inequalities on youth development and protection will then be examined in an attempt to provide a clearer image of the intersection between the two. The article finally intends to derive strategies that allow youth to sustainably participate in reforming their camps, and to establish a flexible urban design prototype which adheres to the needs of the community, addresses its gaps, and enhances youth development and protection in the respective urban space.

## 2. Research methods

To gain a comprehensive and thorough understanding of the formation of Palestinian Refugee Camps in Lebanon since 1948 and their spatial development, historical research is presented. This part of the research relies on an extensive study of the literature found on the formation and development of the camps, including a spatial analysis of several camps. To complement the historical research and have accurate statistics, primary sources such as reports from UNICEF, UNRWA, UNDP and OCHA and were consulted in addition to the secondary sources collected.

Moreover, to explicate on the interrelationship between youth development and spatial configurations, this research entails a case study of Palestinian camps in Lebanon, more specifically Ein El Hilweh Camp. The exclusionary measures imposed against residents of Ein El-Hilweh, reflected through physical barriers such as concrete walls, watchtowers, barbed wires, and Lebanese army checkpoints, along with the physical attributes of spatial elements inside the camp and the strong presence of political organizations, parties, factions, and armed individuals, all contribute to the specific selection of this camp as a case study where youth development is significantly affected. For this purpose, semi-structured interviews were conducted with youth coordinators in local NGOs operating in Ein El Hilweh Camp. This comprised nine individual key informant interviews, and one focus-group discussion with NGO staff operating inside the camp.

The main purpose of the interviews and focus group discussion was to understand the individual and collective factors which shape the ways youth view and dwell the spaces of the camp, how space affects their well-being, the main challenges they face while navigating the spaces of the camp, and the facilities and services they lack which could improve their situation. These were reflected through interviewing Youth Coordinators who are aware of the aforementioned issues given their daily interaction with youth. Conversations with youth coordinators provided comprehensive and inclusive responses to these questions, reflecting the situation of the hundreds of youth in Ein El-Hilweh that they immediately interact with on a daily basis. The interviews were held online between April and May, 2022.

## 3. Palestinian refugees in Lebanon: a political and historical brief

The notion of displacement is quite often portrayed as a transitory state of being, a time of waiting, in which the arrival of refugees and their moment of displacement are conceptualized only as “static in-between statuses” (Brun, 2015). According to Meeus et al. (2019) “[…] this hegemonic view becomes crystallized into provisional statuses and forms of temporary protection or settlement, such as camps,” (Meeus et al., 2019) often portrayed as extreme spaces as their location plays host to the mundane (Stevenson and Sutton, 2011). Hence, this politics of temporariness produces refugee camps with transitory infrastructures and a spatial configuration with defined boundaries, reinforcing its inhabitants’ segregation from the local community, limiting them from engaging in the local of economy, and consequently rendering them dependent on aid agencies for their basic means of survival (Stevenson and Sutton, 2011). As Fawaz (2016) frames it, this is complemented by the legal establishment of the camps on “lands allocated by nation-states in negotiations with global humanitarian organizations to temporarily settle populations fleeing danger until repatriation is possible” (Fawaz, 2016).

Meeus et al. (2019) argue that the arrival of refugees, in fact, evolves in varied forms within the framework of a “lasting temporariness” – rather than ending – where displacement becomes protracted as a result of unresolved conflicts or policies that prevent refugees from returning home, obtaining citizenship, or fleeing to another destination (Meeus et al., 2019). Therefore, this field of political struggle that involves inhabitants, state authorities, international organizations, landowners, political movements, and other factors paves the way for the evolution of temporariness, not into permanence, but rather into permanent temporariness (Diab, 2021). Consequently, this is reflected by the camps’ integration into the urban landscape of the adjacent cities and is challenged by the self-organization of camp settlers “to develop often unexplored urban settings through the social production of space” (Al-Nassir, 2016); however, this does not negate the fact that these spaces are still accompanied by temporary features, as well as, more importantly, by a temporary legal status.

The twelve Palestinian Camps in Lebanon are among concrete examples of “permanent temporary” settlements.^2^ As they remain bound by a sense of temporariness in their status and spatial configurations more than 70 years into their establishment, they continue to catalyze the socio-spatial inequalities experienced by their residents, namely Palestinian Refugees.

### 3.1. The formation of Palestinian refugee camps in Lebanon

The creation of *Israel* and the Arab-*Israeli* war in 1948 resulted in the Palestinian exodus (the Nakba) that led to the displacement of an estimated 750,000 Palestinians to neighboring countries as a result of the *Israeli* occupation’s ethnic cleansing and forcible eviction.^3^,^4^ Mainly fleeing Northern Palestine (Galilee, Haifa, Yafa and other coastal areas), around 130,000 Palestinians took refuge in Lebanon at the time (De Bel-Air, 2012). They gathered around family ties and origins in human settlements initially managed by the Red Cross before UNRWA was established by the end of 1949 to carry out direct relief and work programs for Palestine Refugees in the Near East.^5^ These settlements which were later referred to as Palestinian Refugee Camps were dispersed across the country by the Lebanese state, in a deliberate attempt to exert easier control over Palestinian Refugees (Peteet, 1991). Moreover, Palestinian refugees were indisputably subjected to continuous displacement inside Lebanon, seeking refuge within refuge, as a result of the continuing *Israeli* invasions, the Lebanese civil war, the war of the camps and other massacres whose repercussions were most prominent in the expunction of four Palestinian camps and the recurrent destruction of others (Khawaja, 2011).

### 3.2. The governmental prospect on Palestinian refugees

The Lebanese-Palestinian relationship remains undoubtedly one of the most fragile and complex in the Middle East region. This complexity stems from multiple factors which are either consequent of or serving foreign policies and international political agendas. Not only is the arrival of Palestinians to Lebanon locally challenged, but it is also strongly tied to a foreign perspective vision for Lebanon, affecting its structure and inducing principles of sectarianism in politics and society. The fundamental demographic factor directly impacting this relationship is the sectarian imbalance Palestinian refugees may inflict on the demographics of the modern Lebanese state (Siklawi, 2010). These prevailing notions of sectarianism and classicism pushed the local governing authorities to classify Palestinian refugees by occupation, class, and religion, including naturalization of the majority of Christian Palestinians and wealthy Muslim Palestinians (Siklawi, 2010). This resulted in the horizontal divisions across Palestinian refugees and the marginalization of those belonging to lower classes who were impoverished and placed in camps, fueling them to react against these discriminatory measures.

With the emergence of the Palestine Liberation Organization (PLO) in the early 1960’s onwards, which was later considered the formal representative for Palestinians, political tension was provoked in the region and in Lebanon specifically, with the PLO’s major ramifications being most prominent in the 1967 war as they established military units to fight *Israel*, causing a strident controversy to grow in Lebanon between parties supporting versus parties opposing Palestinian militancy in Lebanon (Siklawi, 2010). Consequently, the Cairo Agreement was signed in 1969 between Lebanon and the PLO, aiming at empowering Palestinians economically, socially, and politically in Lebanon in return of restricting the presence of the PLO to inside the camps (Siklawi, 2010). In addition to Palestinian militant presence, foreign regional intervention played a key role in destabilizing the Palestinian-Lebanese relationship, especially with Jordan’s expansion of PLO operations in Lebanon (Chomsky, 1989; Besson, 1997; Harris, 2006; Siklawi, 2010).

### 3.3. UNRWA’s applied framework in Lebanon and the status of Palestinian refugees

The strategic indifference of the Lebanese government towards refugees, coupled with ongoing political tensions in Lebanon have continued to impede UNRWA’s work in supporting Palestinians in Lebanon for decades now. Besson (1997) suggests that UNRWA’s presence in Lebanon is fundamental amidst the complexity of the governmental prospect on Palestinian refugees, especially that the Lebanese authorities are keen to avoid the integration/naturalization of Palestinian refugees in Lebanese society (Besson, 1997).

Nonetheless, Palestinian refugees in Lebanon are still denied their basic rights, are excluded and marginalized from wider society, and are still strategically kept in a *permanent state of temporariness* by the Lebanese government (Masri, 2020). They are denied access to public social services, including access to healthcare, educational, and emergency services and facilities. Moreover, the vulnerability of Palestinian refugees exacerbates as they are faced with legal restrictions on employment. This serves as the primary obstacle to their integration into the labor market, and remains imposed alongside restrictions to property ownership – ultimately, hindering the possibility for upward social mobility in the country. In addition to the prohibition of Palestinians from owning property as per the amended property law in 2001, they also remain unable to transfer their pre-owned property to their children.^6^ Discrimination against Palestinian refugees remains institutionalized in the amended labor law which bars them from exercising more than thirty-nine syndicated professions (footnote 5). Not only does this reduce employment rates, but it also limits Palestinians’ options to working in more vulnerable, low-skill, informal jobs. Together with these constraints, the decline in adequate educational services serves as a means to increase unemployment and illegal labor. As a consequence of these restrictive measures, Palestinian refugees in Lebanon tend to rely almost entirely on UNRWA as the primary provider of social services and education (Fincham, 2012), supported on field by local and international NGOs which complement its services. This has placed an immeasurable pressure on, and obstructed UNRWA’s programs, transforming the agency’s status from a complementary “temporary” humanitarian aid agency to a service provider (Benoist, 2018).

Today, more than 479,000 refugees are registered with UNRWA in Lebanon (UNRWA, 2020), with 210,000 Palestinian Refugees living in Lebanon including 30,000 from Syria (PRS) (UNICEF, 2022). About 45.4% of those residing in Lebanon live in the country’s twelve refugee camps (Lebanese Palestinian Dialogue Committee, Central Administration of Statistics, Palestinian Central Bureau of Statistics, 2019), in addition to a large population of marginalized Lebanese (UNRWA, 2020). Other nationalities residing in the camps include Iraqis, Egyptians, Sudanese and Bengalis (UNRWA, 2020). Palestinian camps remain overcrowded, with poor housing conditions and deteriorated infrastructure, met by poverty and lack of access to justice (Al Arabiya News, 2022).

Moreover, the Lebanese government’s restrictions imposed on UNRWA’s construction activities limited its ‘Infrastructure and Camp Improvement Program’ to the reconstruction of Nahr El-Bared Camp after its destruction in 2007 and restricted its work to structural rehabilitation of existing shelters and buildings within the officially recognized camps (Knudsen, 2018). This becomes a fundamental challenge as the population residing in the camps continues to increase, ultimately requiring new health, education, and relief buildings (Knudsen, 2018). Moreover, the limited budget UNRWA operates under as well as the restrictions of the government on any attempt of economic independence, have limited the agency’s ability to deliver more sustainable microfinance programs in the country (UNRWA, 2023).

Thus far, Palestinian refugees in Lebanon continue to live in unenviable situations and are “perhaps the most unfortunate and destitute grouping of Palestinian refugees in any Arab host country” (Suleiman, 2006). They are stripped from almost all human and civil rights, and continue to experience spatial, institutional, social and economic marginalization. They have become prolonged refugees who are “confined to camps or segregated settlements where they are partially dependent on humanitarian assistance and often live in dire socio-economic circumstances” (Hanafi et al., 2012). In so, Palestinian refugees are regarded as demographic artifacts in a transient state of being, denied the right of citizenship and any form of decision-making beyond the boundaries of the camps. Accordingly, they are only capable of deploying their (bounded) agency by imposing their imprints on their camps, which “complicates the permanent temporariness of encampment, that opens up a temporality between the permanence of the built (camp) and the temporariness of the political condition (refugeehood)” (Abourahme, 2014). Nevertheless, the demographic weight of Palestinian camps and their economic, social, and political characteristics enabled them to become the genesis of unexpected cities which generally integrate themselves distinctively in urban areas.^7^

As Palestinian refugee camps are “intimately bound up with a temporality of liminality and enduring temporariness,” (Ramadan, 2013) their spatial configuration remains contingent, where the “ever-moving relationship between temporality and materiality” provokes social inequalities (Meeus et al., 2019), specifically when examined within the urban fabric of the cities encircling them. The question of urban configurations thus becomes crucial in examining the inequalities present in Palestinian refugee camps in Lebanon.^8^ These socio-spatial inequalities stem from the geographic characteristics, spatial configurations, the underdeveloped infrastructure, services, and facilities, and they hamper livelihoods through their economic ramifications.

## 4. Socio-spatial inequalities in Palestinian refugee camps in Lebanon

With the concentration of urban poverty and urban marginalization over time, urban segregation becomes inevitable in human settlements characterized by the accumulation of economic, infrastructural, ecological and social deprivation. The inhabitants of these settlements often lack basic facilities, living materials, adequate public spaces, non-defective infrastructure, maintained roads and pavements, and recreational spaces. Moreover, Nowosielski argues that the notion of spatial segregation is most likely to be strengthened by its racial and ethnic character (footnote 7). The temporal dimension characterizing the Palestinian exile to Lebanon, the geographical development of Palestinian refugee camps, and the varying scale of mobility in these settlements allows them to become urban areas, which are isolated and fall under the above mentioned categories. Hence, Palestinian refugee camps in Lebanon are characterized by a “strong local integration” linked to a rapid urbanization of the [host country] parallel to a strong segregation due to the socio-political and legal context (Doraï, 2010a).

### 4.1. Political geographies and boundaries

In what follows, a concrete example is drawn by examining the urban expansion of Shatila Palestinian Refugee Camp. Originally established on an empty lot distant from the city center, the camp managed to integrate itself over the years to become a major part of the urban texture of Beirut. This informal expansion resulted from the demographic pressure on the urbanized camp and the growth of economic opportunities, as refugees from other camps attempted to relocate inside Shatila but were often thwarted by both the government attempting to control the movement of Palestinians between camps, and the residents unwilling to receive newcomers. The extension of Shatila camp is also evident in the aftermath of the 1982 Sabra and Shatila Massacre, which claimed the lives of not only those residing inside the camp but stretched to impact residents of the adjacent neighborhood of Sabra where the camp had sprawled (Peteet, 2005). On the other hand, the geopolitical situation of the camp in the underprivileged suburbs of the city was a major factor to its integration in the city’s “misery belt,” which is defined by Martin (2014) as “an axis of low-income and informal settlements surrounding Beirut’s city center” (Martin, 2014). The implications of poverty and informality on the illegal trades in any human settlement are difficult to overlook. For his part, Halabi (2004) explores the threatened Palestinian moral order by the intensified moral corruption which results from the informal expansion of the area below the poverty line causing a rise of illegal merchants in the camp and Sabra market renowned for drug-dealing, cheap pirated pornographic material, and sex trade (Halabi, 2004). Not only does this continue to threaten women, children and youth, but it also sets the ground for a culture of illegality, ultimately destabilizing the camp’s already troubling security situation. Therefore, as shared by the rest of Palestinian camps in the country, the location of the camps in the suburban informal and underprivileged part of the cities plays a major role in the social inequalities Palestinian refugees face as an implication of the spatial configurations.

In an attempt to isolate the Palestinian camps South of Lebanon as well as Nahr El-Bared in the North, the Lebanese army has placed checkpoints at camp entrances and prevented foreigners from entering the camps without a permit. Moreover, physical boundaries were established by the Lebanese authorities, and were extreme in particular camps such as in Ein El-Hilweh and Mieh Mieh camps, where they were gated with concrete barriers in an attempt to *secure* the area. This left a countless number of Palestinians bounded by walls that segregate them from the city and guards who obstruct their mobility. These walls and guard towers also contribute to “producing a landscape that spatially encodes ethnic segregation between the [Lebanese and Palestinian] communities” (Fincham, 2012).

The Lebanese authorities have aggravated the restrictions on mobility of Palestinian refugees by requesting them to obtain permits to cross into the area monitored by UNIFIL in the South of Lebanon (Hanafi et al., 2012). This restricted the mobility of Palestinians and greatly affected their businesses (Hanafi et al., 2012). These forms of inequality are factors which cause the refugees to remain in the peripheries in areas that confine their main zones of comfort and differences (Fawaz et al., 2009). Therefore, camps organized as strict closed spaces “constitute urban enclaves or satellites located at the urban periphery, lacking in green spaces, and with poor access and poor housing” (Hanafi et al., 2012). The geopolitics of the camp signifies that urban marginalization is strictly entwined with social exclusion – both associated with hierarchical inequalities and a set of excluded rights, duties and obligations to later encompass economic, social and political marginalization as well.

### 4.2. Spatial configurations

The extreme difficulty, and almost impossibility, to own property beyond the boundaries of refugee camps has “squeezed subsequent generations of Palestinian refugees in the confined space that are refugee camps, transforming these areas to slums” (Hanafi et al., 2012), as the majority had no choice between “being trapped in the overpopulated refugee camps deprived of their human right to adequate housing” (Suleiman, 2006), and exiting the camps to rent over-priced apartments where they are at a higher risk of being segregated outside the borders of their *safe spaces.* The consequential overpopulated and informally planned attributes of the refugee camps produce a set of socio-spatial inequalities that distress the residents’ daily lives. The configurations of the camp spaces will be examined at the camp level *(urban planning),* the cluster level *(urban design),* and the dwelling space *(architectural features)* in an attempt to portray subsequent social inequalities as a product of spatial formations.

#### 4.2.1. Urban planning

In 1950, the UN planned a layout of a grid system which divided the camps into defined zones and housing units in its attempt to regulate the scale of the Palestinian camps following the 1948 Exodus and the settlement of Palestinians in various areas in Lebanon. The grid consisted of three-by-four-meter asbestos rooms which were placed in demarcated 100 sqm plots entitled as “right-of-use” for one refugee family (Maqusi, 2017). This division allowed refugees to have ample dwelling spaces within the camp yet was a means of controlling their expansion as they were prohibited from building beyond the demarcated plots which would be considered as spatial violation. However, the absence of private amenities inside the camp pushed refugee families to construct their own inside their plots, thus “[…] rapidly saturating the horizontal plane in the 1960s–70s and initiating horizontal encroachments beyond the plot in the form of *Attabat*” (Maqusi, 2017). As these intrusions became difficult to continue building, vertical stairs were introduced as a new architectural element that facilitated vertical expansions. These alterations produced narrower, irregular streets with random multi-story buildings that jeopardize the security of the camps and interfere with the proper passage of light between buildings. This urban expansion was adopted in almost all camps; however, the specificity of each camp is maintained following its historical, demographic and geographical context. In further depiction of what preceded, the following three case-studies will be presented portraying the variations in urban modifications in correspondence to the former three contexts, respectively, in an effort to examine the social inequalities resulted by spatial configurations.

In the case of El-Buss camp, Palestinian refugees expanded their settlements taking into consideration the historical context. Developed around the former Armenian camp, the first subdivision of the Palestinian camp is composed of terraced three-story houses creating a dense neighborhood occupying a pre-existing urban space with streets that separate houses being narrow and tortuous. As the camp began to expand, the second main subdivision of the camp housed building units that are more spread out, occasionally having a second floor, and some of which have a private garden. However, with the more Palestinian refugees arriving in 1967, and Syrian and Palestinian refugees from Syria post 2011, the camp became overpopulated; however, the distinctions between the two subdivisions can still be made. Therefore, inequalities within the same camp are distinguished throughout its historical expansion, as residents of the less-dense area had wider footprints, more access to green areas, and wider streets that serve as public spaces, in contrast to the overcrowded population that is also closer to the sea, therefore subject to a higher risk of damage and insecurity.

The case of Beddawi camp illustrates the social inequalities generated as the urban planning of the camp evolved with demographic change. Unable to expand horizontally beyond its original plot of land, the camp grew vertically to accommodate the displaced refugees from other Palestinian camps, including “El-Nabatieh camp, destroyed by *Israeli* air raids in 1974; Tel El-Zaatar Camp, razed to the ground in 1976 by Lebanese Christian militias and the Syrian army; and Nahr El-Bared, shelled and bombarded in 2007 by the Lebanese Army in clashes with the Fatah al-Islam militant group” and lately refugees from the ongoing Syrian conflict (Fiddian-Qasmiyeh and Qasmiyeh, 2020). This resulted in narrowing down alleyways, balconies, expanding thresholds and doorsteps, and intertwining clothing lines and electrical cables.

In Burj El-Barajneh, the development of urban planning and the multiple drawbacks of the informal expansion discussed reflect the construction in relation to geographical restrictions. The sloped site of Burj El-Barajneh naturally allowed for informal vertical expansion with minimum provision to pedestrian access with minimal or no setbacks (Samhan, 2008). Moreover, the steepness of the slope posed difficulty in accessibility, which was often facilitated by stairs that neither provide access for disabled persons nor vehicular access. The rise in heights and the conversion of green spaces to buildings in response to the increasing population create dim, insecure and narrow passages which threaten the safety of residents (Feldman, 2015). The development of the urban setting in Burj El-Barajneh Camp leaves its residents with a varied set of inequalities in their everyday life in the camps, including tortuous street patterns and hierarchy of spaces and circulation paths.

#### 4.2.2. Urban design

While the urban design of Palestinian Refugee Camps varies as they are unique in their expansion and planning, they, in principle, suffer from similar inequalities due to the massive urbanization and overpopulation in all camps, and the repercussions of the Syrian war and the Lebanese economic crisis. At the cluster level, the poor conditions are reflected in the lack of visual and physical privacy due to the narrow streetscapes, the clustered house layouts and shared roofs as a result of informal growth, and the absence of public spaces at the ground level. The vertical expansion of the housing units introduced mezzanines and balconies providing very minimal light passages to the streets and minimal distance between opposing units in which residents’ privacies are not fully respected, despite the efforts attempted by residents to shift windows and doors from facing one another – or in some cases building double windows and doors to create a sense of privacy. Moreover, the clustering of space minimizes the possibility of having outdoor spaces in housing units, such as balconies or terraces, which leads families in the same apartment to share the roof of the apartment. This invades the privacy of families in various ways, and also induces gender inequality whereby women are frequently *forced* by conservative husbands to suffocate indoors and not allowed access in the presence of men. Socio-spatial inequalities are thus created as the density of the camps increases, thus intensifying the drawbacks of urban design.

#### 4.2.3. Architectural features

Through examining the smaller scale of the camps, the density of the urban scale seems to be reflected at the dwelling level thus intensifying the former and worsening the living conditions. This is particularly reflected with the increase in population inside the camps as families begin sharing their barely tolerable apartments, which comprise one or two private areas at most. Although the camps’ housing units vary in size, the vast majority of the buildings fall into this category. Moreover, the housing units mostly suffer from poor natural lighting and ventilation which reduces the spatial quality of living and impacts the health of residents due to the lack of proper sanitation and moisture control. Reflecting on the different scales, the development of the spatial configurations in Palestinian Refugee Camps appear to have a major impact in determining the living quality of camp dwellers. Whether on the urban, the cluster, or the architectural level, the formation of spaces in these dense areas have induced inequalities between the Palestinian refugees and neighboring areas, within the camps themselves, and even between dwellers whose *privileges* – in spite of being underprivileged as a collective group at the urban scale – vary in the dwellings that shelter them.

### 4.3. Infrastructure and construction

As UNRWA has been facing the largest financial predicament since its establishment, the hardships faced by Palestinian Refugees has been exacerbating. This is complemented by the increased pressure on the already limited services and infrastructure caused by the increase in population, especially after hosting a large number of Syrians and PRS (Ullrich and Abu-Sharar, 2020). However, the underdevelopment of infrastructure in Palestinian refugee camps are mainly the ramification of multiple restrictions and limitations exercised upon Palestinians by Lebanese authorities. Palestinian Refugee Camps have been considered since their establishment as temporary settlements whose conditions slightly improved over time as the Lebanese authorities allowed the replacement of tents which could not withstand the harsh natural conditions by mud houses with zinc roofs (in an attempt to restrict their vertical expansion). With the arrival of the Palestinian Liberation Organization who began governing the camps in the 1970s, Palestinian refugees were able to improve their living conditions by building more durable concrete units. These units, however, fail to comply with building codes and standards and are not sustainable given their informality, their rapid construction in fear of reversing the right to build, and the dreadful economic conditions of the Palestinian refugees over time that disabled them from acquiring durable construction materials.

The restrictions by the Lebanese government on Palestinian refugees have also complicated the development and enhancement of infrastructure. The Lebanese government strictly forbids the reconstruction of the camps that were entirely demolished by the multiple massacres and invasions which resulted in the displacement of refugees (within their country of refuge) and the overpopulation of camps after four were entirely wiped out. Moreover, the Lebanese army imposes obligatory restrictions on individuals who want to perform any reconstruction inside the Southern camps through a special permit which is difficult and nearly impossible to obtain. This often leads to further investigation by the Lebanese army to verify that building materials are not being smuggled,^9^ mainly through erecting checkpoints adjoining the camp entries. These limitations have been key in the abysmal progress of the camps’ social and physical conditions due to which Palestinian refugees suffer from underdeveloped infrastructure (Sanyal, 2014).

The conditions of the El-Buss, Rashidieh, Burj El-Chemali, Ein El Hilweh and Mieh Mieh camps in the south and Burj El-Barajneh Camp in the suburbs of Beirut therefore deteriorate, where the camps in the south are guarded by the Lebanese Army Forces, and the latter is often monitored by the Internal Security Forces. This leads to the rise in heavily scrutinized living conditions where access to goods, services and employment are further restricted, spatially demonstrated by the presence of checkpoints at camp entrances, as well as by the closure of secondary roads which connect the camp to their surroundings (Sanyal, 2014). This includes extreme shortages of water and electricity which also contribute to the production of dangerous infrastructure. For instance, the configuration of the water and electrical supplies in Shatila’s urbanscape poses continuous threats as water pipes and rain, together with dangling isolated electrical wires, often electrocute many adults and children to death or chronic injuries (Halabi, 2004). The restrictions placed by the Lebanese authorities prevent Palestinian camps from implementing vital developmental projects that improve their despondent conditions; however, they also increase the risk of injury and endanger the lives of those residing the camps, living under roofs prone to collapse during harsh weather conditions.

### 4.4. Facilities and services

Palestinians are still denied access to numerous public social services, especially access to healthcare, educational, and emergency services and facilities. Given that the UNRWA Health Program only delivers primary health care services, and the aid provided to Palestinian refugees to access secondary and tertiary health care services has been decreasing with the shortage of funding, Palestinian refugees suffer from health care that is hardly adequate. Nonetheless, the limited structural and spatial capabilities of the facilities to accommodate the rising number of refugees dependent on Palestinian Red Crescent Society (PRCS) and UNRWA services often result in overcrowded spaces, delayed services, and extreme difficulty in transporting patients, especially with the mobility restrictions in some of the camps. This is met by the absence of tertiary health care which renders Palestinian refugees – often unable to settle their bills in the public or private hospitals providing this type of care – indebted, beholden to parties that finance them, and prone to engage in illegal trades to keep the lives of their families.

Unquestionably, the declining services inside Palestinian camps are further deteriorated by the limited capability to provide high-quality education. The massive number of PRL and PRS children enrolling in only 65 UNRWA schools exceeds the initial capacity of the established schools. Consequently, classes are often overcrowded, in many cases reaching 50 students per class. The limitations of the space do not allow schools inside the camps to entirely include spacious indoor classes and outdoor playgrounds relative to the student capacity, and almost lack any presence of green spaces, all which keep playgrounds heavily occupied with minimal recreational spaces and converts classes into *winterized* indoor playgrounds on rainy days due to the limited sheltered spaces. The implications of such drawbacks are immensely present in increasing dropout rates and illiteracy levels, child labor, early marriage, underqualified (and unqualified) graduates, and other forms of social inequalities which hinder child, youth, and adolescent development – highly consequent of the limitations of the space and its accommodated facilities.

### 4.5. Economic implications of socio-spatial inequalities

The social inequalities provoked by the deterioration of urban configurations present significant economic challenges to the dwellers of these spaces and are further intensified by severe economic implications. These economic implications have most recently shown that the vulnerability of the Palestinian refugees residing in camps could effortlessly be life-threatening. Their safety inside their houses is threatened by the poor infrastructure which in turn is tied to their economic poverty. Their safety is additionally jeopardized due to the fact that their survival is contingent on precarious daily labor in the informal sector. “In essence, the Palestinians in Lebanon were reduced to a form of ‘bare life’ sustained mainly through UNRWA aid” (Sanyal, 2014).

## 5. The interrelationship between youth development and spatial configurations in Ein El Hilweh Camp

Located 3KM south-east of Saida, Ein El-Hilweh Camp is the biggest camp in Lebanon among the 12 officially recognized camps in terms of population, given that its adjacent areas are geographically intertwined within its fabric (ACTED, 2018). It is estimated that approximately one third of Palestinian Refugees in Lebanon reside in Ein El-Hilweh Camp, in the same area which was initially established to accommodate around 9,000 Palestinians (UNRWA, 2018).^10^ The camp’s population has increased over time especially with the displacement of Palestinian Refugees from Tripoli during the civil war and after the 2007 conflict in Nahr El-Bared Camp, and fiercely during the Syrian 2011 war. This increase in population placed a burden on the already overcrowded urban landscape, also heightening the struggle over the limited resources inside the camp.

Similar to all camps, UNRWA still provides essential services (including health care, education, some relief, and social services as well as shelter and infrastructure services) inside the officially recognized camp but does not administer or police the camp; however, adjacent areas are not entirely serviced by UNRWA since they fall beyond the officially recognized boundaries of the camp, and municipal service coverage to these gatherings is limited (ACTED, 2018). UNRWA is not responsible for providing basic urban service – such as water, sewerage, solid-waste management, or road networks – outside the physical boundaries of the camp, and urban services provided by municipalities are “politically difficult, as most residents are living informally, and assistance could be interpreted as a step towards ‘permanent resettlement’” (OCHA, 2017).

The security situation in Ein El-Hilweh is also distressing. It is majorly affected by being a “microcosm of the Palestinian political universe” with all political organizations, parties, and factions in constant tension for leverage, as well as the hideout of fugitives and extremists from the LAF inside the camp (OCHA, 2017). Therefore, recurring incidents of armed conflicts break out inside the camp threatening the safety of the camp residents and hampers their mobility inside the camp, entering and exiting the camp, and their accessibility to services.

These security concerns have led the Lebanese authorities to take extreme measures by constructing an isolation concrete wall with watchtowers and barbed wires leaving no part of the camp unfenced. This wall was thought to promote racism, division, and the culture of estrangement and hatred rather than providing security. Some thought it was tightening the noose on the camps to purge the country from Palestinian refugees (Al Tahhan, 2016). Moreover, the wall built restricts the freedom of movement of people within the camp (especially non-IDs), and from/to the camp, and prevents any integration within the fabric of the city. This affects their protection, mental and physical health, and their access to many services outside the camp. These security measures also hamper businesses activities in the camp as it halts the easy movement of people and products from/to the camp. Moreover, during the construction phase, the Lebanese army installed electronic gates at four entrances of Ein El-Hilweh on June 12th, 2018 (Palestinian Return Centre, 2018). The outrage of Palestinians inside and outside the camp have led the authorities to remove these gates replacing them with multiple checkpoints (Naharnet Newsdesk, 2018).

Moreover, the Lebanese army enforced administrative security restrictions on the entry of construction materials to the camp, requiring camp residents to obtain a permit – which is almost impossible, and thus constraining shelter improvements and infrastructure rehabilitation (Hanafi et al., 2012). Ein El-Hilweh Camp suffers from structural unsafety, where the majority of buildings are affected by moisture problems due to large damp patches and roof leakages. This is heightened by the random expansion of buildings which does not comply with safety standards and threatens the ability of the dire infrastructure to accommodate the population inside the camp. Moreover, Ein El-Hilweh Camp is characterized by road inaccessibility, where 16.4 km of the 22.1 km of roads in the camp are pedestrianized because their width is less than two meters and do not accommodate for 4-wheeled vehicles (this puts patients at serious risks in cases of emergency). Moreover, the estimated surface area of the camp is 0.6 km^2^ as buildings occupy more than 0.3 km^2^ of the total area of the camp – leaving the population density to be around 80,155/km^2^ (UNRWA, 2018). This results in small and overcrowded shelters, poor living conditions including lack of privacy and quietness, and over-burdened infrastructure. Moreover, this demographic pressure along with the urban situation demonstrates a serious concern in terms of environmental health.

Palestinian refugees are among the marginalized population who are suffering the most from the ramifications of the economic crisis happening in Lebanon and exacerbating since 2019 with the devaluation of the Lebanese Lira, crisis in the banking sector and restricted access to the money, increased employment rates, deflated wages, and a hyperinflation in livelihood. The effect of the pandemic amidst this situation has been to increase the unemployment rate amongst Palestinian workers from 60 to 90% (Nasreddine, 2020). This also coincided with UNRWA’s financial crisis caused by Trump’s decision to cut the entire US budget in addition to donor countries failing to meet their commitments which caused a massive scarcity in services provided by UNRWA.

This difficult situation resulted in a large increase in drop-out rates among youth due to economic poverty and limited employment opportunities. The economic situation in addition to the increased hardships with the arrival of almost 6,000 Palestinian Refugees from Syria also caused an increased use of drugs, also leading to increasing levels of violence (Nilsson and Badran, 2019). This is exacerbated with the misguidance of youth and the limited resources for other forms of expression. The camp has limited recreational spaces for youth, and the concrete wall and barbed wires have cut through countless buildings, rooftops, compounds, and empty spaces.

## 6. Findings

The findings from the individual interviews and the FGD suggest that socio-spatial inequalities are immensely present in Ein El-Hilweh Camp and are primarily affecting children and youth. The sectors of youth protection, capacity and skills building, and participation are characterized with relatively weak provision and are intertwined with the dire conditions of spatial configurations inside the camp and the adjacent areas.

The architectural and urban setting inside the camp and adjacent areas – including all its urban elements: buildings, roads and infrastructure contribute negatively to the development of youth in a tridimensional manner. First, the main architectural factors contributing to the weakened protection of youth are characterized by overcrowded, dim and narrow streets which decrease the visibility of verbal, physical or sexual harassment, abuse, or violence. In addition, the limited personal space inside the houses leads to domestic violence. Moreover, these urban conditions ease substance dealing and substance use. Another important factor which affects the protection of youth is the numerous vehicles in narrow streets. Second, the main architectural factors contributing to the weakened capacity and skills building of youth are the presence of unhealthy spaces youth resort to, such as cybercafés, coffee shops and the extension of shops and cafes to the street. Third, the main architectural factors contributing to the weakened participation of youth are the strong presence of political offices, as well as the aforementioned elements which contribute to the unsafety of the camps.

On the other hand, the absence of space is an equal factor which affects youth development. The safety elements which enhance youth protection such as youth protection networks, youth protection centers, safe spaces for youth at-risk (especially females) are either not strongly present inside the camp or are few with limited access. Moreover, the absence of safe spaces and youth friendly spaces forces youth to resort to other unhealthy facilities, such as the streets, coffee shops, cybercafés, or restricts some of them from going out of their neighborhood circle. Youth are also challenged by the lack of adequate infrastructure which fails to cater for the overpopulation, and the lack of spaces to intervene in – which is an obstruction to any attempt at participating in spatial rehabilitation or construction.

These factors which are key contributors to the underdevelopment of youth in Ein El-Hilweh are also met by the definition or control over spaces, which affects the feasibility or accessibility of youth to facilities and services. The political dominance over the spaces mostly determines “who gets what” and “who goes where,” which prevents a large number of youth from receiving specific services, leads to dispute or armed conflict, and provokes hatred and rage among youth. Moreover, the definition of spaces discourages many parents from sending their children, mainly females, to NGOs/CSOs and other recreational spaces inside the camp.

The security measures taken by the Lebanese authorities including the concrete wall besieging the camp, the barbed wires, the checkpoints at the main entrances of the camp, the smaller checkpoints at multiple small entrances of the camp, and the watchtowers encircling the camp exacerbate the situation of youth protection, capacity and skills building, and participation. The absence of an easy and quick evacuation mechanism threatens the lives and well-being of youth in incidents where they are trapped in armed conflict, followed by an individual or a group of individuals, as well as incidents where their health is at-stake. Moreover, youth are unable to resort to facilities outside the camp due to the long queues at the checkpoints, the closure of the camp due to multiple security incidents, and the harassment faced at the checkpoints. The enclosure of the camp is also a prime factor to the inability of spatial horizontal expansion and integration with the fabric of the city, which affects youth in multiple ways. Recreational facilities and youth friendly spaces are very difficult and nearly impossible to build. In addition, the communication and knowledge exchange between youth inside Ein El-Hilweh and youth outside the camp and adjacent areas is minimal unless with former residents in the camp. This form of isolation creates a cultural barrier between youth inside and outside, inducing a sense of fear and protection for cultural exchange from both sides. Moreover, youth’s spatial perception of the camp as an imprisoning space besieged with a concrete wall similar to the ones *Israelis* build increases their desperation, demotivation, and hopelessness. This situation also drives a modest amount of youth to engage in acts of mischief inside the camp. Therefore, not only does the suppressed physical freedom of youth affect their accessibility to services and facilities, but also alters their perception of space and inclusion, induces feelings of otherness, and affects their well-being. The geopolitical characteristics of the camp and adjacent areas, as well as the view of the Lebanese authorities on Palestinian Refugees in Lebanon are key contributors to the socio-spatial inequalities and the hindered development of youth inside the camp and adjacent areas.

Not only are the spatial factors which affect the development of youth bound by their form or structure, but also in the ways in which they are defined (politically, religiously), occupied, and used. The function of the space has equal importance as its form in the pool of youth development. The transformation of mosques and a few NGO/CSO centers into spaces for political recruitment, the transformation of coffee shops and cybercafés into drug-dealing areas, the transformation of streets into smoking hubs, political offices, and places for armed individuals, are all examples of how spaces may become unsafe when they divert from their initial functions and gain threatening attributed functions. Moreover, the enclosing forms of architectural elements may be used to obscure their function.

Further to the abovementioned factors which contribute to the underdevelopment of youth in Ein El-Hilweh, the population density affects the provision of enough services for youth and many fall behind as a result of inaccessibility, nepotism, political affiliation, and other forms of prioritization. Moreover, urban poverty and social inequalities are a major obstacle when it comes to youth development. The majority of youth are engaged in securing their livelihoods rather than building their skills and capacities, participating in volunteering and non-income generating activities. This demotivation is also met by fear of retaliation or hopelessness due to the political dominance over services, facilities, and decision-making process which make youth feel enervated to participate in their community.

While both males and females are subjected to harassment, abuse or violence, victims of sexual harassment, abuse, or violence are mostly females, while victims of verbal and physical violence are mostly males. Moreover, individuals who do not conform to a certain gender norm are victims of all kinds of harassment, abuse, or violence. On another hand, female youth are expected to remain at home, and thus their main areas of interaction are limited to their homes, their friends’ homes, or the roofs of the apartments of their families, friends, or neighbors during summer. Male youth are less societally restricted and thus in addition to the aforementioned, their main areas of interaction are outdoors, including cafes or coffee shops, their neighborhoods, but mostly on the streets smoking *nargile.* Male youth have more freedom to explore the space inside (and outside) the camps, while the spatial experience of female youth is more constrained. This segregation in space occupancy embeds an element of unsafety, especially when female youth are walking alone in the presence of male youth sitting in front of shops and houses. The spatial restriction of females is also a consequence of the lack in safe spaces for females, or the lack of a safe route to these spaces. In addition, the public participation of female youth is obstructed with cultural and societal traditional perceptions on females, especially in positions of power, and thus acts as an obstacle to their engagement and participation in youth committees, neighborhood committees, inhabitant committees and popular committees. This is especially the case because the current authorities inside the camp are older men with a traditional mindset. Moreover, female participation in activities and volunteering campaigns is at many times met with objection and assault because of the nature of physical activities such as cleaning or planting. The nature of activities female youth participate in are linked to the cultural perception of the way females are to occupy spaces. Therefore, the encounters of youth generally vary according to their gender, and with female youth being the most vulnerable. This variation impedes the development of female youth in a more accelerated manner and puts them at risk.

Moreover, the socio-spatial experiences of youth with disabilities seem to be the most vulnerable and severe amongst youth in Ein El-Hilweh Camp. They are faced with multiple challenges including navigation of space, accessibility to services and centers, and a demeaning social stigma which affects their natural development negatively and worsens their vulnerable situation in Ein El-Hilweh. Non-IDs (and many Palestinian Refugees from Syria) in Ein El-Hilweh are among the most vulnerable group in terms of spatial experiences as they are unable to “cross borders” at the checkpoints, and their accessibility to facilities and services inside and outside Ein El-Hilweh remains very difficult, and at many points impossible.

On another note, the situation of youth in Ein El-Hilweh Camp is in the first place a situation they do not have control over. The characteristics of Ein El-Hilweh are determined by historical, political and sectarian factors, the repercussions of the civil war, cultural barriers and traditions, and a massive economic crisis – all which current generations are suffering from and have nothing to do with. Youth do not have a choice in the places they wish to go to or the activities they perform. The activities they are engaged in and the spaces they occupy is mainly due to the absence of other alternatives. While the activities performed by youth differ based on the different factors discussed, the majority of youth are not constructive or productive in their free time but are rather engaged in a very trite routine. This also embeds the culture revolving around these activities, which hamper the societal view of youth, their view of themselves, and most importantly entrenches a stigmatized role for youth females that obstructs their development.

However, building the skills and capacities of youth, and providing them with a protected environment to participate in decision-making processes largely expands the possibility of change. The role of youth, who are mostly updated with the advancement of this generation, and who are mostly opposes of the deep-rooted cultural barriers, is crucial in reducing the socio-spatial inequalities in Ein El-Hilweh Camp. Moreover, the exposure of youth to “the outside” is a driver for positive change. Finally, the solutions and strategies proposed are not radical, but their implementation is capable of reducing the socio-spatial inequalities youth face in Ein el-Hilweh and is therefore capable of fostering youth development by enhancing their protection, increasing their capacity and skills building, and encouraging their participation. The proposed strategies rely on funding, advocacy, and a supporting entity to encourage their implementation.

## 7. Discussion

The intersectionality between youth development and spatial configurations in Palestinian camps is majorly corresponsive in the same direction – in that both youth development and spatial configurations affect one another either positively or negatively. The spatial configurations of Palestinian Camps in Lebanon are generally negative contributors to the development and protection of youth inside the camps. Palestinian camps suffer from high levels of urban poverty as they face exclusion and deterioration in architectural elements and in infrastructure services and facilities. Youth have limited spaces to engage in constructive and participatory activities, and their safety and security is constantly prone to be threatened. The dire conditions of the urban setting inside the camp act as an obstruction towards the protection, capacity and skills building, and participation of youth thus hindering their development.

However, in the presence of fostered youth protection and capacity and skills building, youth engagement and participation in the modification of their spaces act as essential drivers of change which contribute to the reduction of urban poverty and to the development of urban strategies that can sustain their development and provide an incubating environment for them to grow during the stages of their youth and beyond. Therefore, the case study of Ein El-Hilweh confirms the suggested hypotheses which indicate that youth protection and development are more likely to be deteriorated when the spatial configurations in which they reside are not adequate, and that the urban development trajectory is more likely to be sustainable and inclusive, and urban poverty is most likely to be reduced, when youth, who receive necessary capacity building, are provided the opportunity to engage in the planning and design process.

Youth protection, capacity and skills building, and participation are cross-cutting areas of intervention which are co-dependent for youth development. Therefore, any programming for youth needs to ensure the provision of the three for a comprehensive and cyclical approach. Moreover, programming should also involve parents who are vital actors in implementing social and behavioral change in the community, thus providing a growth-friendly environment for their children to develop, and for their youth to transition into constructive adulthood. It is essential that a collaborative approach including all actors in Palestinian Camps in Lebanon (NGOs/CSOs, UNRWA, UNICEF, UNDP, popular committees, security committees, inhabitant committees, Political Organizations, parties, and factions, parents, children and youth) is taken. Moreover, it is important to intensify the support to the most vulnerable groups of youth in Palestinian camps: female youth, youth with disabilities, and non-IDs. Youth are drivers of change in the community, and an utmost effort needs to be exerted to ensure that this group of individuals are empowered to enhance their communities.

The development of youth is hindered by the lack of space and presence of space. The urban setting of Palestinian Camps in Lebanon plays a significant role in reducing the levels of protection, and obstructing capacity and skills building and the opportunities for youth engagement and participation. Urban planning and design inside the camps is crucial to improve the living conditions of its residents. This includes infrastructure refinement, traffic congestion control, urban expansion control, shelter and building rehabilitation, optimization of indoor and outdoor spaces to the best interest of children and youth – including youth-friendly spaces, green roofs, green walls, green areas, recreational areas, and cultural centers. This is complemented by ensuring that all residents – including the most vulnerable, are able to access and benefit from these facilities.

The geopolitics of the camps are essential factors contributing to their dire conditions. Therefore, a comprehensive attempt at urban refinement needs to include an effort to liaise with the Lebanese authorities to reduce the strict security measures imposed on the camps which halts the mobility of numerous individuals inside the camp and encourages the presence of wanted individuals. It also restricts attempts at rehabilitation and new construction. It is important to note that the security situation in Palestinian Camps is part and parcel of that outside them, as these areas remain part of the cities they are located in. The integration of Palestinian camps within the fabric of the city is essential to improving the livelihood of Paletinians, the economic cycle of the host state, fostering healthy communication between all residents in Lebanon, and to reducing the levels of discrimination and segregation against Palestinian Refugees in Lebanon.

Urban planning and design should also be embedded in a manner which empowers and engages the community in the process, improves their knowledge on socio-spatial inequalities, and embeds the culture of change. With the deteriorated economic situation, youth volunteerism and youth-led initiatives seem to be less feasible unless supported by NGOs, CSOs, or International Organizations. The urban poverty inside Palestinian Camps in Lebanon is worsening, leading the majority of youth to either drop out of education or fill their free time securing their livelihoods. Activities that are not income-generating become the least priority for the majority of youth who are unable to leave their work, despite the social impact of these activities on their community. Therefore, income-generating programs are essential for youth, especially those who drop out of education, to ensure that they are securing their livelihoods in safe and non-abusive ways. Moreover, designing these programs to induce urban refinement is key to reducing both social and spatial inequalities.

The interrelationship between social inequalities and spatial inequalities is inevitable. However, in a context of urban poverty, the spatial aspect cannot be tackled unless the social culture of urban refinement is induced in the community. Moreover, it is also crucial to address the importance of political stability inside the camp to ensure that socio-spatial inequalities are addressed in a non-obstructive environment, and that youth development is fostered with fewer challenges. This requires an enforcement of law to ensure and maintain the security and safety of the Palestinian Camps in Lebanon. In the absence of state actors, UNRWA is perceived by Palestinian Refugees in Lebanon as the reference state which provides them with primary services and responds to their needs. With the current financial crisis UNRWA is facing, and its anticipated continuity – if not worsening, NGOs and CSOs with the support of IOs and donors are trying to fill a huge gap left by the downsizing of UNRWA’s services. However, NGOs and CSOs do not have the capacity or accountability to replace UNRWA – just as UNRWA, despite its continuous effort, is also bound by the hosting state. In the current situation, Palestinian Refugees in Lebanon and Palestinian Camps in Lebanon are entirely dependent on non-state actors as they are faced with continued temporality.

## 8. Concluding remarks

In examining the operation of the politics of temporariness in Palestinian Refugee Camps, it is evident that there is a violent opposition by the Lebanese authority towards a permanent resettlement or implantation of Palestinians in the country, translating into restrictive policies regarding social, economic and civil rights of the Palestinians, often violating the Universal Declaration of Human Rights, and exerting “intense demographic and economic pressure on the limited space of the camps themselves” (Sanyal, 2014). Whether the position on resettlement is rejected by Palestinians who insist on the right of return whereby the camps are political claims of return is not examined in this paper; however, with second, third, fourth and fifth generations being born in Lebanon in a span of 74 years, the sense of belonging varies between a romanticized thought and an ardent reality.

Refugee camps have demonstrated that they have become *de facto* urban centers given their demographic and social weight, despite their de jure prohibition. Where boundaries between the cities and the camps are indistinguishable, space plays a critical role in the articulation of rights among refugees – especially youth. The Lebanese authorities embed restrictive measures on the daily lives of Palestinians in the effort to keep the spaces in which they dwell marginalized and segregated, attempting to socially exclude Palestinians and deny them the ability to integrate into the economic activity of the country around them. The Palestinian camps are both one with and yet remain separate from the urban environment. Therefore, socio-spatial inequalities are present in refugee camps and are produced by the geographic politics of the camps, their spatial configurations, and the underdevelopment of infrastructure, services and facilities in these camps. The camps and refugees also suffer the economic implications of the socio-spatial inequalities thus produced.

The settlement of refugees could extend to traverse the expected temporality and their final destination could be bound by their countries of origin, their initial host countries, or other host countries. It is crucial to challenge the notion of refugees as only waiting to become citizens (again). It is important to signal that to support refugees is to recognize the fluidity of temporariness as a dynamic process. To empower refugees must mean granting them civil and economic rights, recognizing the transnational character of their identity, and radically improving the urban conditions of their space (Hanafi, 2008). Therefore, they should be considered “actors who contribute, through their initiatives and coping strategies, to the development of the cities that host them” (Doraï, 2010b), and the plan of managing refugees must “strengthen their own resources and self-reliance and avoid creating dependency” (Stevenson and Sutton, 2011).

It is also crucial to note that the rehabilitation of a space does not contradict with the anticipated desire of or right to return. The nature of refugees as dynamic individuals in space and time, means that they are able to dwell within differences of temporalities without detaching from their initial space of reference. Instead, it is vital that these refugees maintain their dignity and the ability to acquire decent living standards by integrating into the host societies as productive individuals sharing the responsibility of maintaining the development of their host country. Most importantly, this emphasizes being granted the right to participate in developing the urban fabric where they settle in ways that ensure their development and protection, and accordingly leads to a cohesive developed urban sprawl into the cities of the host community which reduces the isolation of spaces inhabited by refugees and the socio-spatial inequalities that emerge as a consequence. Moreover, this includes contributing to the economy of the host community at a multispectral level by engaging in the labor market and increasing productivity. Only in doing so are refugees able to improve both their social as well as spatial conditions and reduce socio-spatial inequalities.

Palestinian Refugee Camps in Lebanon demonstrate an exemplary case of permanent temporariness. The dire conditions of Palestinian Refugees in Lebanon and the Palestinian Refugee Camps have been exacerbating as Palestinians have been living in temporary conditions since 1948, paying the price of international, regional and local politics. They are neither able to live in settling conditions or attain a settling status in Lebanon, nor able to leave Lebanon - becoming permanent refugees. This temporality is the prime reason for their situation – namely in the course of this study, it hindered youth development and deteriorated urban conditions. Despite the loopholes and entry points to enhance the situation of Palestinian Refugees in Lebanon, the continued temporality imposed by the state on Palestinian Refugees and the continued exclusionary measures which are worsening their livelihoods make it extremely difficult for any form of mending to the situation. Therefore, any attempt at reducing the socio-spatial inequalities in Palestinian Camps in Lebanon is a temporary solution unless Palestinians in Lebanon are no longer considered by the state to be more than a transient influx of individuals waiting for return, but rather an integrated community who has access to social, economic, and civil rights, and most importantly a right to the city. Further in-depth research could explore each factor contributing to the deterioration of urban space or the hindering of youth development through in-depth exploration. Moreover, to provide a more comprehensive methodology, interviews with NGOs/CSOs, UNRWA, UNICEF, UNDP, popular committees, security committees, inhabitant committees, Political Organizations, parties, and factions, parents, and most importantly – youth, could be conducted as they are all main actors involved in the situation in Palestinian camps.

## Data availability statement

The raw data supporting the conclusions of this article will be made available by the authors, without undue reservation.

## Ethics statement

The studies involving humans were approved by the Institutional Review Board (IRB) at the Lebanese American University. The studies were conducted in accordance with the local legislation and institutional requirements. The participants provided their written informed consent to participate in this study.

## Author contributions

YE-Z led on research conceptualization and design, data collection, and the ethical review process. YE-Z and JD co-wrote this paper. JD led on the editing and submission processes. All authors contributed to the article and approved the submitted version.

## Footnotes

^1^The camp is located in the Darb Es-Sim, Mieh Mieh, Saida Ed-Dekermane municipality, part of the Saida District and South Governorate.

^2^There are currently 12 Palestinian camps hosting over 50% of the almost half a million refugees: four in and near Beirut (Burj al-Barajne, Shatila, Dbayeh, and Mar Elias), two in Tripoli (Beddawi and Nahr al-Bared), three near Tyre (Burj Shemali, El-Bass and Rashidiyeh), two near Saida (Ein el-Helwe and Mieh Mieh) and one near Baalbek (Wavel).

^3^Lebanon currently includes 12 Palestinian Refugee Camps across the country, with four camps in Beirut and its suburbs (Burj El Barajneh Camp, Shatila Camp, Mar Elias Camp, and Dbayeh Camp), two camps in the North governorate (Nahr El-Bared Camp and Beddawi Camp), one camp in the Bekaa area (Wavel Camp), two camps in Saida area (Ein El-Hilweh Camp and Mieh Mieh Camp), and three in Tyre district (Burj El-Chemali Camp, El-Buss Camp and Rashidieh Camp). During the civil war and the war with Israel, Palestinian camps suffered immensely, especially in Beirut and suburbs, and the South of Lebanon. Three camps (Tal El Zaatar Camp and Jisr El Basha Camp in Beirut and Nabatieh Camp in the South) were destroyed, and a fourth camp (Gouraud Camp in Baalbek) was evacuated.

^4^The Nakba and Palestine Refugees | IMEU Questions and Answers. https://imeu.org/article/the-nakba-and-palestine-refugees-imeu-questions-and-answers

^5^UNHCR was established roughly 1 year after UNRWA was founded in response to the European Refugees post World War II.

^6^Lebanon’s third periodic state party report states: The issued property law (Decree 296) in May 2001, prohibits Palestinian from the ownership of property and deprives them of the right to transfer their already purchased apartments and deeds to their children. The Lebanese government considers the Law in harmony with its opposing stance to the resettlement of Palestinian refugees in Lebanon.

^7^For example, Nahr El-Bared Camp was a business trade hub in the North for Palestinians and non-Palestinians and it is highly believed by the Palestinian community that its destruction was an attempt to break its positive sprawl into the city and the integration of Palestinians.

^8^Gatherings adjacent to these camps are deprived from infrastructure projects and improvement programs, given that they do not fall under UNRWA’s mandate and receive minimal to nil aid from the municipalities responsible for their maintenance.

^9^These include cement, rods, sand, tiles, aluminum, paint materials, wood and iron doors and windows, glass panels, water pipes, electrical wires, water tanks, as well as generators.

^10^The adjacent areas are: Al Ta’meer Al Tahtany, Al Sikkeh, Al Taware’e, Baraksat, Bustan Abu Jameel, Bustan Al Quds and Uzo, Jabal al-Haleeb, and Fadlo Wakim.
